# Reciprocal regulation of CIP2A and AR expression in prostate cancer cells

**DOI:** 10.1007/s12672-022-00552-8

**Published:** 2022-09-13

**Authors:** Hao-Wen Chuang, Jian-Hua Pan, Yi-Xuan Cai, Darius Rupa, Ting-Syuan Huang, Tzu-Chien Kuo, Chiao-Wen Lin, Chi-Wei Chen, Chia-Chin Lin, Herng-Sheng Lee, Ta-Chun Yuan

**Affiliations:** 1 Department of Pathology and Laboratory Medicine, Kaohsiung Veterans General Hospital, No. 386, Dazhong 1st Rd, Zuoying Dist, Kaohsiung, 813414, Taiwan, ROC; 2 Department of Life Science, National Dong Hwa University, No. 1, Sec. 2, Da Hsueh Rd., Shoufeng, Hualien, 974301 Taiwan, ROC

**Keywords:** Prostate cancer, CRPC, CIP2A, AR, PLK1

## Abstract

Cancerous inhibitor of protein phosphatase 2A (CIP2A) is an oncoprotein overexpressed in human malignancies, including prostate cancer (PCa). In this study, we aimed to explore the oncogenic function of CIP2A in PCa cells and its underlying mechanism. We showed that 63.3% (38/60 cases) of PCa tissues exhibited a high CIP2A immunostaining, compared to 25% (3/12 cases) of BPH samples (p = 0.023). Furthermore, the protein level of CIP2A was positively correlated with patients’ short survival time and nuclear AR levels in PCa tissues. Compared to PZ-HPV-7, an immortalized prostate cell line, androgen-sensitive LNCaP C-33, androgen-independent LNCaP C-81, or 22Rv1 cells exhibited a high CIP2A level, associated with high protein and phosphorylation levels of AR. While AR expression and activity modulated CIP2A expression, manipulating CIP2A expression in PCa cells regulated their AR protein levels and proliferation. The reduction of CIP2A expression also enhanced the sensitivity of PCa cells toward Enzalutamide treatment. Our data further showed that depletion of polo-kinase 1 (PLK1) expression or activity in C-81 or 22Rv1 cells caused reduced protein levels of c-Myc and AR. Notably, inhibition of PLK1 activity could abolish CIP2A-promoted expressions in c-Myc, AR, and prostate-specific antigen (PSA) in C-33 cells under an androgen-deprived condition, suggesting the role of PLK1 activity in CIP2A-promoted AR expression. In summary, our data showed the existence of a novel regulation between CIP2A and AR protein levels, which is critical for promoting PCa malignancy. Thus, CIP2A could serve as a therapeutic target for PCa.

## Introduction

Prostate cancer (PCa) is the most commonly diagnosed cancer among men in the developed world. Like normal prostate cells, most PCa cells require androgens for their growth and survival. Thus, androgen deprivation therapy (ADT) which aims to reduce androgen levels or block the activity of androgen receptor (AR) becomes the standard treatment for locally advanced or metastatic PCa. Most tumors initially respond to ADT but later are refractory to hormonal therapies. Within 2–3 years, the diseases eventually develop the castration-resistant PCa (CRPC), resulting in tumor relapse and cancer-related death [1]. Until recently, therapeutic options for CRPC are limited and provide a minimal increase in overall survival. Thus, it is urgently needed to develop suitable therapies for patients with CRPC.

The AR is a steroid hormone receptor that functions as a ligand-regulated transcription factor. Upon activation by androgens, AR dimerizes as a homodimer and subsequently binds to androgen-responsive elements (ARE) on target genes, activating gene expression for regulating the growth and survival of prostate epithelial cells. However, deregulated AR portein level or activity plays a key role in the development of CRPC. Accumulated evidence demonstrates that the majority of CRPC is still dependent on the AR signaling and exhibits a reactivated AR even under castration. Numerous mechanisms have been identified to drive the progression from androgen-dependent to CRPC [2]. Interestingly, many studies have shown that progression of CRPC is associated with a high AR protein level. However, only 10–20% of CRPC exhibits *AR* gene amplification [3]. It indicates that increased AR protein level in CRPC may be due to the mechanism other than gene amplification, such as activated transcription factors. Besides, multiple signaling pathways that activate different protein kinases promote AR transcriptional activity by serine/threonine phosphorylation [4].

CIP2A, encoded by the *KIAA1524* gene, is an oncoprotein overexpressing approximately 39 to 90% of malignant tissues. Its protein level is correlated with aggressive disease and the poor survival rate [5]. Knockdown of CIP2A expression in various cancer cells inhibits the growth of xenografted tumors [6, 7]. Accumulated evidence indicates that CIP2A executes its oncogenic function by suppressing the phosphatase activity of protein phosphatase 2A (PP2A). CIP2A could directly bind to PP2A and inhibition of CIP2A leads to enhanced PP2A activity [6]. Thus, CIP2A promotes the activities of various kinases and transcription factors by keeping them away from PP2A-mediated dephosphorylation and degradation. It is well-characterized that CIP2A interacts with c-Myc to prevent PP2A activity toward c-Myc serine 62, stabilizing c-Myc against degradation [6]. Furthermore, CIP2A regulates polo-like kinase 1 (PLK1) stability and activity, facilitating cell-cycle progression and tumor development [8]. In PCa cells, CIP2A is highly expressed in patient specimens of hormone-naïve PCa and CRPC [9, 10]. Knockdown of CIP2A expression in LNCaP cells led to reduced cell viability and colony formation [9]. However, the role of CIP2A in facilitating castration resistance in PCa cells and its underlying molecular mechanism is mostly unknown.

In this study, we investigated the novel regulatory mechanism between CIP2A and AR in PCa cells. We showed that positive correlation exhibited between CIP2A and AR expression in PCa tumors. We further provided evidence that AR signaling could transcriptionally regulate CIP2A protein levels. Importantly, CIP2A modulated AR protein level required the activity of PLK1. Our data demonstrated the oncogenic role of CIP2A in promoting AR protein level, which potentially serves as a therapeutic target for CRPC treatment.

## Materials and methods

### Antibodies and chemicals

Antibodies against CIP2A (lot number K2420), PP2Ac (lot number D2309), or β-actin (lot number J0421) and horseradish peroxidase (HRP)-conjugated secondary antibodies were from Santa Cruz Biotechnology (Dallas, Texas, USA). Antibodies against PSA (Lot number 3), PLK1 (lot number 6), phospho-c-Myc (Ser62) (lot number 5), or c-Myc (lot number 15) were purchased from Cell Signaling (Danvers, MA, USA). Antibodies against phospho-AR (Ser81) (lot number 3497306) or AR (lot number 3256650) were obtained from Millipore (Burlington, MA, USA). Enzalutamide was obtained from MedChemExpress (Monmouth Junction, NJ, USA). LB100 and BI6727 were purchased from BioVision (Milpitas, CA, USA). Okadaic acid, TD52, Bicalutamide, and other chemicals were obtained from Sigma-Aldrich (Burlington, MA, USA).

### Cell culture

Cell culture media, FBS, charcoal/dextran-treated FBS (c-FBS), and supplements were purchased from ThermoFisher Scientific (Waltham, MA, USA). Immortalized prostate cell line PZ-HPV-7 cells, androgen-sensitive LNCaP (passage 28), and androgen-independent 22Rv1 prostate carcinoma cell lines were purchased from the Biosource Collection and Research Center (BCRC, Hsinchu, Taiwan) and cultured as described previously [11]. Cells were fed twice and split once weekly with trypsinization defined as one passage. LNCaP cells with passage numbers less than 33 were designated as LNCaP C-33. The passage numbers of LNCaP cells over 80 were designated as LNCaP C-81 [12], obtained from Dr. Ming-Shyue Lee at National Taiwan University (Taipei, Taiwan). To reduce androgen effects on cell culture, a steroid-reduced (SR), i.e., phenol red-free RPMI-1640 medium supplemented with 5% charcoal/dextran-treated FBS, 1% glutamine, and 0.5% gentamicin, was used.

### Construction of KIAA1524 expression vector and lentiviral infection

For constructing the *KIAA1524* expression vector, the full-length human *KIAA1524* cDNA flanked by *att*L sequences and cloned in the pENTR223.1 vector was purchased from MyBioSource (San Diego, CA, USA). Using the Gateway LR Clonase II enzyme mix kit (ThermoFisher Scientific; Waltham, MA, USA), the *KIAA1524* cDNA was shuttled from the pENTR223.1 vector to the *att*R-contained pLX302 lentiviral vector (Academia Sinica; Taipei, Taiwan). For gene overexpression or knockdown experiments, the production or infection of vector-contained lentiviruses was performed as described previously [13].

### Real-time PCR

Total RNA content was isolated from cells using an RNA purification kit (Geneaid; Taipei, Taiwan), and cDNA was synthesized using a GoScript reverse transcription system (Promega; Madison, WI, USA), following the manufacturer’s protocols. All real-time PCR reactions were conducted by the Bio-Rad CFX96 Real-time PCR Detection System (Hercules, CA, USA). The amplification was conducted using the iQ SYBR Green Supermix (Bio-Rad). The thermal cycling conditions were 30 cycles (94 °C for 1 min, 60 °C for 1 min, and 72 °C for 2 min). The paired primers used for AR and GAPDH were AR (forward): 5′-CCTGGCTTCCGCAACTTACAC-3′; AR (reverse): 5′-GGACTTGTGCATGCGGTACTC-3′; GAPDH (forward): 5′-TGGTATCGTGGAAGGACTCATGAC-3′; and GAPDH (reverse): 5′-TGCCAGTGAGCTTCCCGTTCAGC-3′. These primers were designed according to the database of RTPrimerDB [14].

### Cell proliferation assay

The virus-infected or inhibitor-treated cells with a density of 0.7–1 × 10^5^ cells/well were seeded onto 24-well plates. After incubation for 48 h, one set of attached cells was harvested and counted as day 0. The remaining cells were fed with fresh medium, and the cell number was counted on day 3.

### Cell lysis and immunoblotting

The experimental procedure was described previously [13]. Briefly, an aliquot of cell lysates in the SDS-PAGE sample buffer was separated by electrophoresis and then transferred to a nitrocellulose membrane for immunoblotting. The membrane was then blocked with 5% nonfat milk in TBST and incubated with primary antibodies overnight at 4 °C. After rinsing, the membrane was incubated with HRP-conjugated secondary antibodies for 1 h at RT. The specific protein was then detected by an ECL reagent kit (PerkinElmer; Waltham, MA, USA).

### Patient samples

Tissue samples, including 12 benign prostatic hyperplasia (BPH) and 60 PCa, were collected from patients who underwent prostatectomy and whose personal information was de-identified between 2003 and 2006 at Kaohsiung Veterans General Hospital (Kaohsiung, Taiwan). All specimens from the archival files and reviewed by two experienced pathologists. None of the patients had received chemotherapy, hormonotherapy, or radiotherapy before surgery. The study protocol was approved by the Institutional Review Board of Kaohsiung Veterans General Hospital (#KSVGH21-CT5-01) in accordance with the IRB’s guidelines and regulations.

### TMA construction and immunohistochemistry

The tissue microarrays (TMA) were prepared from the formalin-fixed paraffin-embedded tissue blocks using a manual TMA Arrayer MTA-1 (Beecher Instruments; Sun Prairie, WA, USA). Immunohistochemical (IHC) procedures were conducted following the manufacturer’s protocol of the Bond III Autostainer (Leica Biosystems; Wetzlar, Germany). Briefly, tissue sections were deparaffinized and rehydrated. Subsequently, the slides were immersed in ethylenediaminetetraacetic acid buffer (pH 9.0) for 40 min. The sections were then incubated with an anti-CIP2A (clone 2G10; lot number NB100-74663, Novus Biologicals, Littleton, CO, USA) at 1:100 dilution or an anti-AR (clone AR27; lot number 6067897, Leica Biosystems, Wetzlar, Germany) at 1:50 dilution at RT for 30 min. The signal was amplified using a BOND Polymer Refine Detection kit (Leica Biosystems). The protein levels of CIP2A and nuclear AR in the prostate epithelia were evaluated independently by two experienced pathologists who scored using a semi-quantitative H-score method, which considered both the staining intensity and the percentage of positively stained cells. The intensity of the staining was divided into three grades: negative (0), weak (1+), moderate (2+), and strong (3+). The percentage (0 to 100%) of staining was determined and a total score ranging from 0 to 300 was obtained [15].

### Statistical analysis

The MedCalc version 19.1.6 (MedCalc Software; Ostend, Belgium) and PRISM version 5.0 (Graphpad Software; San Diego, CA, USA) were used for statistical analyses. A median cut-off immunoscore value of 160 was used to separate cases into low and high CIP2A immunoexpression. A Student’s two-tailed t-test or a one-way ANOVA was applied to determine the significance between groups. Fisher’s exact or chi-square test was applied to evaluate associations between categorical variables. Spearman’s or Pearson’s correlation was used to assess the correlation between variables. The Kaplan–Meier method was used to analyze overall survival (OS), and the log-rank test was used to compare groups. A p*-*value of < 0.05 was considered significant.

## Results

### The level of CIP2A is correlated with AR protein in PCa tumors

**Fig. 1:**
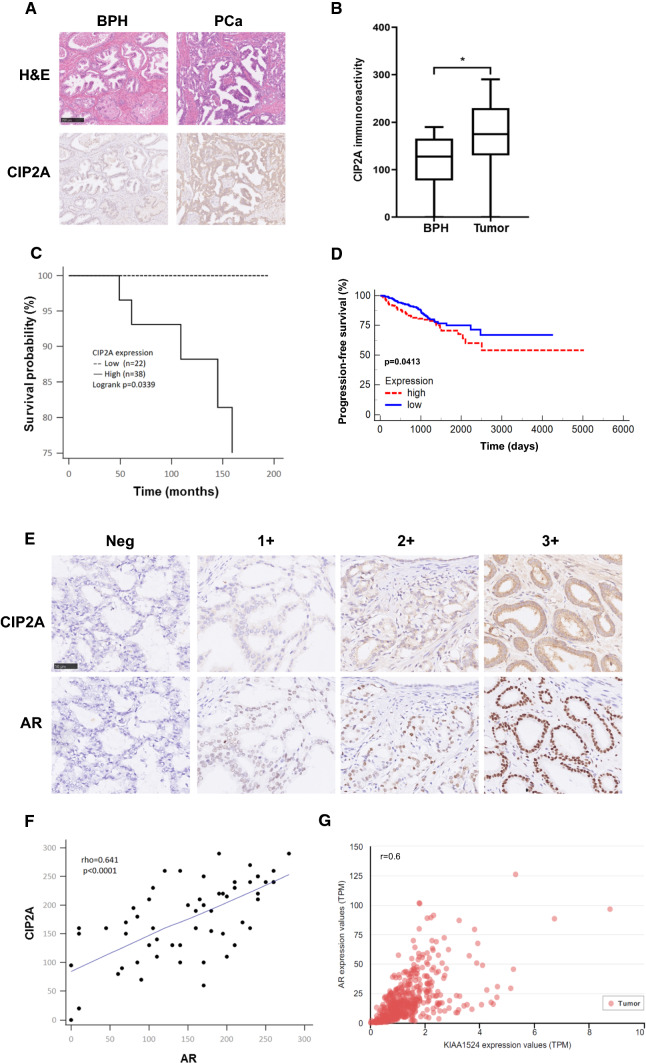
Association between CIP2A and AR protein levels in PCa tumors. **A** Hematoxylin–eosin (H&E) and cytoplasmic CIP2A immunostaining in the BPH (1 +) or PCa (3 +) specimen. Scale bar 250 μm. **B** Differential protein level of CIP2A in BPH (n = 12) and PCa tissues (n = 60) (p = 0.0113). **C** Kaplan–Meier survival curve in subgroups of PCa patients with high (solid line) or low (dash line) level of CIP2A (p = 0.0339). **D** Kaplan–Meier analysis of Progression-free survival (PFS) based on clinical and molecular data for prostate cancer patients (TCGA Prostate Cancer, PRAD). The patients were stratified by the protein levels in their tumors of *KIAA1524* (p = 0.0413). **E** The consistency of CIP2A and nuclear AR immunoreactivities in the same tissues. The representative images of negative (Neg), weak (1+), moderate (2+), or strong (3+) immunostaining for CIP2A and AR. Scale bar, 50 μm. **F** Positive correlation between CIP2A and AR protein level in 60 PCa specimens. Spearman’s correlation coefficients (rho) = 0.641, p < 0.0001. **G** Data were downloaded from the UALCAN database. Pearson’s correlation analysis showed gene expression of CIP2A and AR genes in the TCGA database (Prostate Cancer, PRAD). Pearson’s correlation coefficient (r) = 0.6

We initially examined CIP2A protein level in PCa tissue arrays containing 12 BPH and 60 PCa specimens. The representative images showed that the PCa tissue exhibited cytoplasmic CIP2A immunopositivity in the epithelial cells with a strong (3+) staining, whereas the BPH sample only showed a weak (1+) immunoreactivity (Fig. 1A). Figure 1B showed that CIP2A level was significantly higher in the PCa tissues than in BPH specimens (p = 0.023). Among 60 PCa tissues, 57 cases (95%) exhibited CIP2A immunopositivity, and 38 cases (63.3%) displayed high CIP2A immunoexpression (Table 1). Table 2 summarizes the intensity data of CIP2A immunostaining in arrayed tissues. Despite no significant associations with pT stage, lymph node metastasis, lymphovascular and perineural invasion, or PIN, CIP2A level was significantly associated with Gleason score in PCa tumors (p = 0.032). Furthermore, high CIP2A immunoscore was significantly correlated with a short survival time (p = 0.0339, log-rank test) (Fig. 1C). Similarly, results from the TGCA database (http://xena.ucsc.edu/) showed that the PCa patients with a high expression of the *KIAA1524* gene with a low ratio of progression-free survival (PFS) (p = 0.0413) (Fig. 1D). We further determined the correlation between CIP2A and nuclear AR protein level in arrayed PCa tissues. The representative images in Fig. 1E showed the consistency of CIP2A and AR immunoreactivities in the same tissues, and the scatter plot in Fig. 1F indicated a moderate positive correlation between CIP2A and nuclear AR level (rho = 0.641, p < 0.0001). Results from the TGCA database (http://ualcan.path.uab.edu/index.html) further indicated a moderate positive correlation between the *KIAA524* and *AR* genes in PCa samples with the Pearson correlation coefficient (r) = 0.6 (Fig. 1G) [16]. The data collectively suggested that CIP2A was highly expressed in PCa tissues, and its level was associated with AR protein in PCa tumors.

**Table 1:** Expression of CIP2A in BPH and PCa tissues

	n (100%)	Low (%)	High (%)	*p* value
BPH	12	9 (75)	3 (25)	0.023*
PCa	60	22 (36.7)	38 (63.3)	–

*BPH* benign prostatic hyperplasia, *PCa* prostate cancer

^*^p < 0.05

**Table 2:** Expression of CIP2A expression and clinicopathological parameters in PCa tissues

Characteristics	n (100%)	Low (%)	High (%)	*p* value
Gleason score				0.032*
6	16	2 (12.5)	14 (87.5)	
> 6	44	20 (45.5)	24 (54.5)	
pT stage				0.285
pT2	31	9 (29.0)	21 (71.0)	
pT3-4	29	13 (44.8)	16 (55.2)	
LNMets				1.000
pos	3	1 (33.3)	2 (66.7)	
neg	57	21 (36.8)	36 (63.2)	
LVI				0.102
pos	12	7 (58.3)	5 (41.7)	
neg	48	15 (31.2)	33 (68.7)	
PNI				0.766
pos	45	16 (35.6)	29 (64.4)	
neg	15	6 (40.0)	9 (60.0)	

*LNMets* lymph node metastasis, *LVI* lymphovascular invasion, *PNI* perineural invasion

^*^p < 0.05

### The expression and activity of AR modulated CIP2A expression

**Fig. 2:**
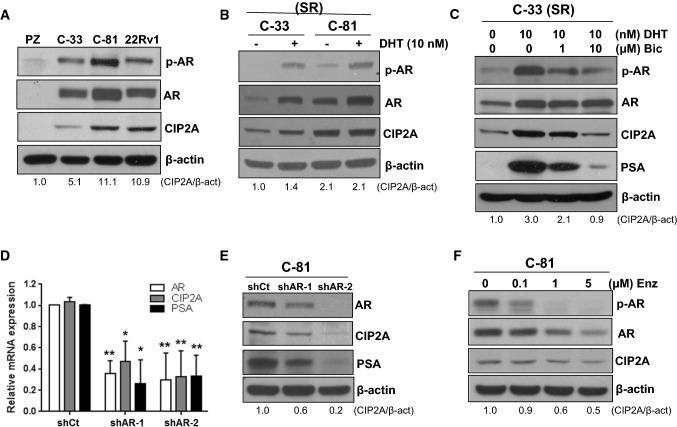
AR signaling modulated CIP2A protein level in PCa cells. **A** Total lysates from PZ-HPV-7 (PZ), LNCaP C-33, C-81, and 22Rv1 cells are used for immunoblotting with antibodies against the phosphorylation level of AR at Ser81 (p-AR) and the levels of full-length AR and CIP2A. **B** LNCaP C-33 or C-81 cells are cultured in a steroid-reduced (SR) medium for 48 h and are then fed with fresh media containing 10 nM dihydrotestosterone (DHT). After additional 48 h of incubation, total lysates are prepared for immunoblotting. **C** LNCaP C-33 cells are cultured in an SR medium for 48 h and are then fed with fresh media containing 10 nM DHT without or with 1 μM or 10 μM bicalutamide (Bic). After 48 h of incubation, total lysates are prepared for immunoblotting. **D** LNCaP C-33 or **E** C-81 cells are infected with viruses carrying the *LacZ*- or *AR*-targeted shRNA (i.e., shCt, shAR-1, or shAR-2). The cell lysates are prepared for real-time PCR or western blot analyses. Data are expressed as the mean ± SD from three independent experiments. Each set of experiments is conducted in duplicate. *p < 0.05, **p < 0.01 vs. the ratio of the corresponding shCt control cells. **F** LNCaP C-81 cells are incubated with 0.1 μM, 1 μM, or 5 μM of Enzalutamide (Enz) for 24 h. Subsequently, the total lysates are prepared for immunoblotting. The protein level of CIP2A is quantified using ImageJ software and the relative ratio is normalized to the β-actin level

Next, we examined whether AR signaling could regulate CIP2A protein levels. We analyzed the protein or phosphorylation levels of AR and CIP2A in an immortalized prostate cell line PZ-HPV-7 (PZ), an androgen-sensitive LNCaP C-33 (C-33), and two androgen-independent PCa cell lines, i.e., LNCaP C-81 (C-81) and 22Rv1 [12, 17]. Compared to PZ-HPV-7 cells, C-33, C-81, and 22Rv1 cells exhibited higher protein or phosphorylation levels of AR and CIP2A. Moreover, the protein or phosphorylation levels of AR and CIP2A in C-81 cells were much higher than those in C-33 cells (Fig. 2A). Under a steroid-reduced (SR) condition, C-81 cells exhibited higher protein or phosphorylation levels of AR and CIP2A than C-33 cells. Treatment of 10 nM DHT greatly enhanced the protein levels of AR and CIP2A in C-33 cells but did not affect the level of CIP2A in C-81 cells (Fig. 2B). The stimulating effects of DHT on the protein levels of CIP2A and prostate-specific antigen (PSA) in C-33 cells can be abolished by bicalutamide, an antiandrogen, following a dose-dependent manner (Fig. 2C). Results from the quantitative real-time PCR analyses showed that knockdown of AR expression in C-33 cells caused significant decreases in the mRNA levels of AR, CIP2A, and PSA, suggesting the functional role of AR in the transcriptional regulation of CIP2A expression (Fig. 2D). The knockdown of AR expression in C-81 cells also caused decreases in the protein levels of CIP2A and PSA (Fig. 2E). Treatment of Enzalutamide, a second-generation nonsteroidal antiandrogen, in C-81 cells resulted in reduced AR protein and phosphorylation levels, correlating with decreased CIP2A and PSA in a dosage-dependent manner (Fig. 2F). These results indicated that AR played a critical role in regulating CIP2A protein level in PCa cells.

### The expression of CIP2A regulated AR expression and cell proliferation

**Fig. 3:**
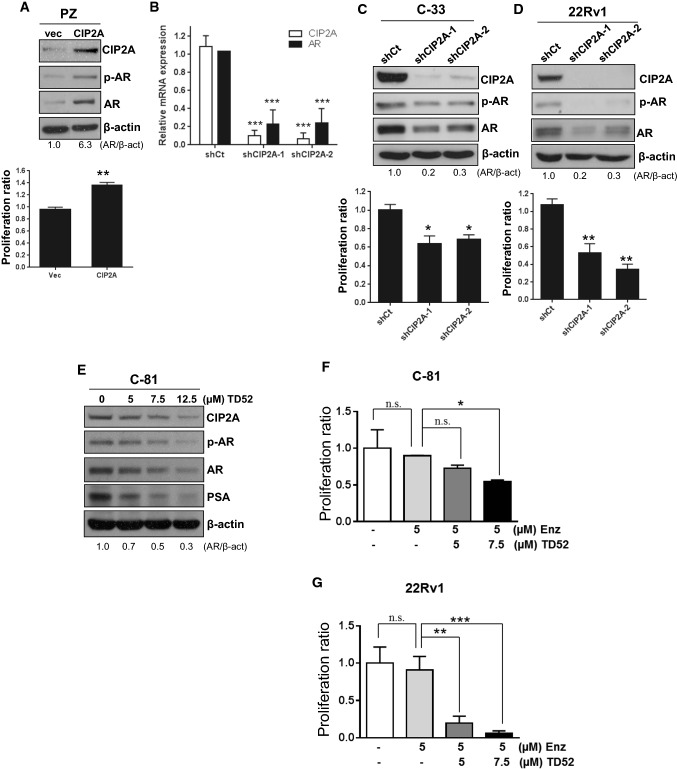
Effects of CIP2A expression on the AR protein level and proliferation in PCa cells. **A** The vector-or CIP2A-expressed PZ-HPV-7 (PZ) cells are cultured for western blot analyses (upper panel) and cell proliferation assays (lower panel). **B** LNCaP C-33 cells are infected with viruses carrying the *LacZ*- or *KIAA1524*-targeted shRNAs (i.e., shCt, shCIP2A-1, or shCIP2A-2). The total lysates are prepared for real-time PCR. **C** LNCaP C-33 and **D** 22Rv1 cells infected with viruses carrying the shCt, shCIP2A-1, or shCIP2A shRNA are prepared for western blot analyses (upper panel) or cell proliferation assays (lower panel). **E** LNCaP C-81 cells are incubated with 5 μM, 7.5 μM, or 12.5 μM of TD52 for 24 h. Subsequently, the total lysates are prepared for immunoblotting. The level of AR is quantified using ImageJ software and the relative ratio is normalized to the β-actin level. **F** C-81 or **G** 22Rv1 cells are treated without or with 5 μM Enzalutamide (Enz) in the absence or presence of 5 μM or 7.5 μM of TD52. After 72 h of incubation, cells are harvested for cell proliferation assays. Data are expressed as the mean ± SD from three independent experiments. Each set of experiments is conducted in duplicate. *p < 0.05, **p < 0.01 ***p < 0.001 vs. the ratio of the corresponding control cells. *ns* not significant

We next examined whether CIP2A could regulate the AR protein level and cell proliferation in PCa cells. As shown in Fig. 3A, an increased level of CIP2A in PZ-HPV-7 cells led to elevated AR protein and phosphorylation and increased cell proliferation. Knockdown of CIP2A expression in C-33 cells caused significant decreases in AR mRNA levels (Fig. 3B), suggesting the role of CIP2A in the transcriptional regulation of AR expression. In addition, CIP2A-knockdown C-33 or 22Rv1 cells exhibited diminished AR protein levels and reduced cell proliferation (Fig. 3C, D). We further treated C-81 cells with different dosages of TD52, a CIP2A inhibitor [18]. As shown in Fig. 3E, the treatment of TD52 caused decreased CIP2A levels, correlating with reduced levels of AR and PSA in a dose-dependent manner. We further examined whether reducing the CIP2A protein level might enhance the sensitization of C-81 or 22Rv1 cells to Enzalutamide. As shown in Fig. 3F and G, the treatment of Enzalutamide alone did not affect cell proliferation. However, the co-treatment of Enzalutamide and TD52 led to significant decreases in cell proliferation. These data suggested that CIP2A could modulate PCa cells in AR protein level, cell proliferation, and sensitivity toward antiandrogen treatment.

### Decreased PP2A activity or expression caused reduced levels of c-Myc and AR

**Fig. 4:**
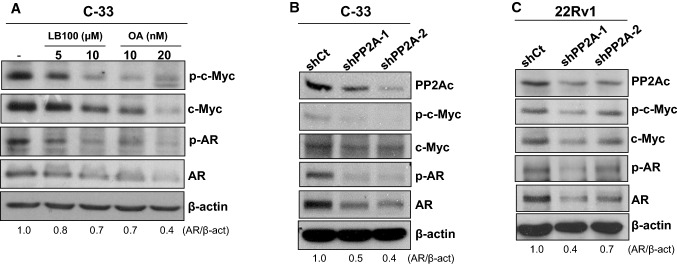
Effects of inhibiting or knocking down PP2Ac on the protein levels of c-Myc and AR in PCa cells. **A** LNCaP C-33 cells are incubated with 5 μM or 10 μM of LB100, or 10 nM or 20 nM of okadaic acid (OA) for 24 h. Subsequently, the total lysates are prepared for immunoblotting. **B** C-33 or **C** 22Rv1 cells infected with viruses carrying the *LacZ*-targeted shRNA (shCt) or *PPP2CA*-targeted shRNAs (shPP2A-1 or shPP2A-2) are harvested for western blot analyses

Because CIP2A has been shown to inhibit PP2A, thus promoting the stability and activity of c-Myc [6], a transcription factor involved in modulating AR gene expression [19, 20], we speculated that CIP2A-promoted AR level was mediated by suppressing PP2A activity. Surprisingly, the inhibition of PP2A activity in C-33 cells by treating cells with LB100, a specific PP2A inhibitor [21], or okadaic acid (OA), a nonspecific PP1 and PP2A inhibitor [22], was unable to enhance the protein or phosphorylation levels of c-Myc and AR. In contrast, it caused decreased protein and phosphorylation levels of c-Myc and AR, following a dose-dependent manner (Fig. 4A). We further knocked down the expression of PP2A in C-33 or 22Rv1 cells. As shown in Fig. 4B and C, knockdown of PP2Ac, the catalytic subunit of PP2A, caused reduced protein or phosphorylation levels of c-Myc and AR. Our data clearly showed that inhibition of PP2A activity or protein level failed to promote c-Myc or AR phosphorylation and protein levels. Thus, the CIP2A-promoted AR level was independent of PP2A inhibition.

### PLK1 was involved in CIP2A-promoted AR level

**Fig. 5:**
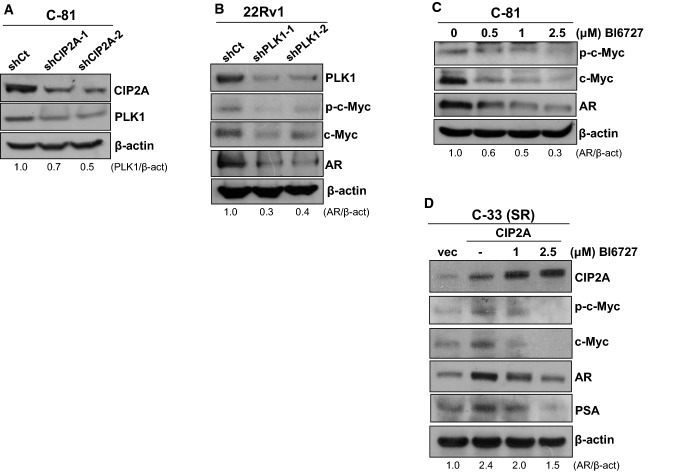
Effects of PLK1 expression or activity on the CIP2A-regulated AR protein level. **A** The total lysates from the shCt control and CIP2A-knockdown C-81 cells were prepared for western blot analyses. **B** 22Rv1 cells infected with viruses carrying the *LacZ*-targeted shRNA (shCt) or *PLK1*-targeted shRNAs (shPLK1-1 or shPLK1-2) were harvested for western blot analyses. **C** C-81 cells were treated with 0.5 μM, 1 μM, or 2.5 μM of BI6727. Cells treated with an equal volume of solvent (DMSO) served as the control. After 24 h of incubation, the cell lysates were prepared for immunoblotting. **D** The CIP2A-overexpressed C-33 cells were cultured in steroid-reduced (SR) media and treated without or with 1 μM or 2.5 μM of BI6727 for 24 h. The vector-expressed cells (vec) served as the control. Total cell lysates were then prepared for western blot analyses

We next examined the role of polo-like kinase 1 (PLK1), a serine/threonine kinase, in mediating the CIP2A-promoted AR protein level. As shown in Fig. 5A, the knockdown of CIP2A expression in C-81 cells led to a decrease in the PLK1 protein level, suggesting the role of CIP2A in regulating the PLK1 level. We further examined whether PLK1 could regulate c-Myc or AR protein levels in PCa cells. As shown in Fig. 5B, knockdown of PLK1 expression in 22Rv1 cells resulted in reduced phosphorylation or protein level of c-Myc and AR. Moreover, treatment of BI6727 (volasertib), a PLK1 inhibitor [23], led to decreases in the protein and phosphorylation levels of c-Myc and AR, following a dose-dependent manner (Fig. 5C). We next examined whether PLK1 is required for CIP2A-modulated AR level. Results from Figure 5D showed that increased CIP2A protein levels in C-33 cells caused increases in the protein and phosphorylation levels of c-Myc, AR, or PSA under an androgen-depleted condition. Notably, CIP2A-promoted effects were abolished by BI6727 treatment following a dose-dependent manner. These results indicated that CIP2A could modulate PLK1 protein level or activity, which was required for the CIP2A-promoted AR level.

## Discussion

CIP2A is a well-known oncoprotein in various cancers. Its oncogenic function in modulating castration resistance in PCa cells has not been studied. In this study, we showed a positive correlation between CIP2A and AR protein levels in PCa tumors. While AR signaling could modulate CIP2A expression, CIP2A reciprocally regulated AR levels in mRNA and protein levels. Importantly, our data showed the CIP2A-modulated AR level required the activity of PLK1. To our knowledge, this is the first report showing the novel regulation between CIP2A and AR protein levels in PCa cells, which may contribute to the castration resistance of PCa cells.

Based on the TGCA database, no difference is found in the expression of *KIAA1524* gene between normal and PCa samples. However, lines of evidence indicated high CIP2A protein levels in PCa tumors, correlating with high Gleason scores [10, 24]. In concordance with the findings, our data showed the high CIP2A level in PCa tumors compared to BPH tissues, which was significantly associated with high Gleason scores. Importantly, our results showed that CIP2A level was positively correlated with AR protein level in PCa tumors. The cell-line study supported this notion that CIP2A protein level was higher in PCa cells than immortalized prostate cells, correlating with their AR levels. Interestingly, the level of CIP2A in LNCaP C-81 cells and 22Rv1, two androgen-independent cell lines, is even higher than that in androgen-sensitive C-33 cells, similar to the observation in clinical specimens [9]. The high CIP2A level in advanced PCa might be due to activated AR signaling. Indeed, Khanna et al. [9] first showed that increased AR protein level and activity enhance CIP2A expression in LNCaP cells. In contrast, knockdown of CIP2A expression in LNCaP or VCaP cells leads to reduced CIP2A levels. Our data further revealed that AR protein level was critical for the mRNA expression of CIP2A. Although the detailed mechanism of how AR regulates CIP2A requires further investigation, overexpressed or activated AR in CRPC cells indirectly activates the function of transcription factors, such as ETS1 [25, 26], ELK1 [25], and E2F1 [27], to interact with the CIP2A promoter region and enhanced its expression. Alternatively, increased protein level or activated AR could directly bind to CIP2A intronic region and regulate CIP2A expression [9].

AR gene amplification or increased protein level is commonly found in CRPC patients [28]. Increased AR level in hormone-sensitive cells enhances their resistance to the antiandrogens [29]. Our data showed that CIP2A could regulate AR at mRNA and protein levels. In addition, changes in CIP2A protein level affected the response of PCa cells toward androgens or antiandrogens. These results might be caused by the fact that the CIP2A-promoted AR level sensitized the PCa cells to respond to a lower concentration of androgens [30]. However, the critical question is how CIP2A regulates AR levels. Although studies have suggested that PP2A negatively regulates AR activity or protein level [31, 32], our data clearly showed that depletion of PP2A activity or protein level caused reduced AR level instead of enhancing its protein level. This notion is supported by the observation that treatment of okadaic acid in LNCaP and 22Rv1 caused decreases in their AR levels [33]. Thus, the CIP2A-promoted AR level in PCa cells was mediated by a PP2A-independent mechanism.

Our results clearly showed that CIP2A could modulate PLK1 protein levels in immortalized prostate and PCa cells. The functional linkage between CIP2A and PLK1 was further supported by the observation that CIP2A interacts with the polo-box domain of PLK1, which maintains PLK1 stability and enhances its kinase activity [8]. PLK1 is highly expressed in PCa tissues and is linked to higher-grade tumors [34]. Treatment of PLK1 inhibitor caused reduced AR protein level and inhibited tumor growth in LNCaP CRPC xenografts [35, 36]. In vitro kinase assays further showed that PLK1 binds to c-Myc and phosphorylates it at serine 62, promoting c-Myc protein stability [37]. In agreement with these findings, our data showed that PLK1 silencing or inhibitor treatment in C-81 or 22Rv1 cells caused reduced protein or phosphorylation levels of c-Myc and AR. Furthermore, CIP2A-promoted levels in c-Myc, AR, and PSA were abolished by BI6727 treatment under an androgen-deprived condition, suggesting that PLK1 was required for CIP2A-promoted AR level. Since CIP2A could regulate AR at the transcriptional level, it is possible that CIP2A promoted PLK1 stability and activity, which in turn enhanced c-Myc transcriptional activity and caused increased AR gene transcription. Alternatively, CIP2A-activated PLK1 may enhance AKT activity and leads to AKT-mediated Twist1 phosphorylation and nuclear accumulation. The activated Twist1 further increases AR expression through binding to E-boxes in the AR promoter [38].

In summary, we have demonstrated that CIP2A was highly expressed in PCa tumors, associated with AR protein level. While AR signaling could upregulate CIP2A level in an AR-dependent manner, CIP2A inversely modulated AR level. The underlying mechanism was not mediated by PP2A inhibition but was associated with PLK1 activity. Thus, our data supported the oncogenic role of CIP2A in promoting AR expression and castration-resistant growth in PCa cells, potentially serving as a therapeutic target for CRPC treatment.

## Data Availability

The data sets used and/or analyzed during the current study are available from the corresponding author upon reasonable request.

## Abbreviations

PCa: Prostate cancer
BPH: Benign prostatic hyperplasia
CRPC: Castration-resistant prostate cancer
AR: Androgen receptor
CIP2A: Cancerous inhibitor of protein phosphatase 2A
PP2A: Protein phosphatase 2A
PLK1: Polo-like kinase 1
RT: Room temperature

## References

[1] Imamura Y, Sadar MD (2016). Androgen receptor targeted therapies in castration-resistant prostate cancer: bench to clinic. Int J Urol.

[2] Huang Y, Jiang X, Liang X (2018). Molecular and cellular mechanisms of castration resistant prostate cancer. Oncol Lett.

[3] Shiota M, Yokomizo A, Naito S (2011). Increased androgen receptor transcription: a cause of castration-resistant prostate cancer and a possible therapeutic target. J Mol Endocrinol.

[4] Koryakina Y, Ta HQ, Gioeli D (2014). Androgen receptor phosphorylation: biological context and functional consequences. Endocr Relat Cancer.

[5] Khanna A, Pimanda JE (2016). Clinical significance of cancerous inhibitor of protein phosphatase 2A in human cancers. Int J Cancer.

[6] Junttila MR, Puustinen P, Niemela M (2007). CIP2A inhibits PP2A in human malignancies. Cell.

[7] Come C, Laine A, Chanrion M (2009). CIP2A is associated with human breast cancer aggressivity. Clin Cancer Res.

[8] Kim JS, Kim EJ, Oh JS (2013). CIP2A modulates cell-cycle progression in human cancer cells by regulating the stability and activity of Plk1. Cancer Res.

[9] Khanna A, Rane JK, Kivinummi KK (2015). CIP2A is a candidate therapeutic target in clinically challenging prostate cancer cell populations. Oncotarget.

[10] Vaarala MH, Vaisanen MR, Ristimaki A (2010). CIP2A expression is increased in prostate cancer. J Exp Clin Cancer Res.

[11] Chang YL, Hsu YK, Wu TF (2014). Regulation of estrogen receptor alpha function in oral squamous cell carcinoma cells by FAK signaling. Endocr Relat Cancer.

[12] Igawa T, Lin FF, Lee MS (2002). Establishment and characterization of androgen-independent human prostate cancer LNCaP cell model. Prostate.

[13] Hsiao YH, Huang YT, Hung CY (2016). PYK2 via S6K1 regulates the function of androgen receptors and the growth of prostate cancer cells. Endocr Relat Cancer.

[14] Pattyn F, Speleman F, De Paepe A (2003). RTPrimerDB: the real-time PCR primer and probe database. Nucleic Acids Res.

[15] Miyamoto KK, McSherry SA, Dent GA (1993). Immunohistochemistry of the androgen receptor in human benign and malignant prostate tissue. J Urol.

[16] Chandrashekar DS, Bashel B, Balasubramanya SAH (2017). UALCAN: a portal for facilitating tumor subgroup gene expression and survival analyses. Neoplasia.

[17] Sramkoski RM, Pretlow TG, Giaconia JM (1999). A new human prostate carcinoma cell line, 22Rv1. In Vitro Cell Dev Biol Anim.

[18] Yu HC, Hung MH, Chen YL (2014). Erlotinib derivative inhibits hepatocellular carcinoma by targeting CIP2A to reactivate protein phosphatase 2A. Cell Death Dis.

[19] Grad JM, Dai JL, Wu S (1999). Multiple androgen response elements and a Myc consensus site in the androgen receptor (AR) coding region are involved in androgen-mediated up-regulation of AR messenger RNA. Mol Endocrinol.

[20] Lee JG, Zheng R, McCafferty-Cepero JM (2009). Endothelin-1 enhances the expression of the androgen receptor via activation of the c-myc pathway in prostate cancer cells. Mol Carcinog.

[21] Wei D, Parsels LA, Karnak D (2013). Inhibition of protein phosphatase 2A radiosensitizes pancreatic cancers by modulating CDC25C/CDK1 and homologous recombination repair. Clin Cancer Res.

[22] Dounay AB, Forsyth CJ (2002). Okadaic acid: the archetypal serine/threonine protein phosphatase inhibitor. Curr Med Chem.

[23] Yim H (2013). Current clinical trials with polo-like kinase 1 inhibitors in solid tumors. Anticancer Drugs.

[24] Celikden SG, Baspinar S, Ozturk SA (2020). CIP2A expression in high grade prostatic intraepithelial neoplasia and prostate adenocarcinoma: a tissue microarray study. Malays J Pathol.

[25] Pallai R, Bhaskar A, Sodi V (2012). Ets1 and Elk1 transcription factors regulate cancerous inhibitor of protein phosphatase 2A expression in cervical and endometrial carcinoma cells. Transcription.

[26] Khanna A, Okkeri J, Bilgen T (2011). ETS1 mediates MEK1/2-dependent overexpression of cancerous inhibitor of protein phosphatase 2A (CIP2A) in human cancer cells. PLoS ONE.

[27] Laine A, Sihto H, Come C (2013). Senescence sensitivity of breast cancer cells is defined by positive feedback loop between CIP2A and E2F1. Cancer Discov.

[28] Hu R, Denmeade SR, Luo J (2010). Molecular processes leading to aberrant androgen receptor signaling and castration resistance in prostate cancer. Expert Rev Endocrinol Metab.

[29] Chen CD, Welsbie DS, Tran C (2004). Molecular determinants of resistance to antiandrogen therapy. Nat Med.

[30] Waltering KK, Helenius MA, Sahu B (2009). Increased expression of androgen receptor sensitizes prostate cancer cells to low levels of androgens. Cancer Res.

[31] Bhardwaj A, Singh S, Srivastava SK (2011). Modulation of protein phosphatase 2A activity alters androgen-independent growth of prostate cancer cells: therapeutic implications. Mol Cancer Ther.

[32] McClinch K, Avelar RA, Callejas D (2018). Small-molecule activators of protein phosphatase 2A for the treatment of castration-resistant prostate cancer. Cancer Res.

[33] Chen S, Kesler CT, Paschal BM (2009). Androgen receptor phosphorylation and activity are regulated by an association with protein phosphatase 1. J Biol Chem.

[34] Weichert W, Schmidt M, Gekeler V (2004). Polo-like kinase 1 is overexpressed in prostate cancer and linked to higher tumor grades. Prostate.

[35] Zhang Z, Chen L, Wang H (2015). Inhibition of Plk1 represses androgen signaling pathway in castration-resistant prostate cancer. Cell Cycle.

[36] Shin SB, Woo SU, Yim H (2019). Cotargeting Plk1 and androgen receptor enhances the therapeutic sensitivity of paclitaxel-resistant prostate cancer. Ther Adv Med Oncol.

[37] Ren Y, Bi C, Zhao X (2018). PLK1 stabilizes a MYC-dependent kinase network in aggressive B cell lymphomas. J Clin Invest.

[38] Zhang Z, Hou X, Shao C (2014). Plk1 inhibition enhances the efficacy of androgen signaling blockade in castration-resistant prostate cancer. Cancer Res.

